# Cross-Talk Between Large Artery Stiffness and Retinal Microvasculature in Children: The ExAMIN Youth SA Study

**DOI:** 10.3389/fped.2021.795301

**Published:** 2021-12-16

**Authors:** Yolandi Breet, Ashleigh Craig, Wayne Smith, Shani Botha-Le Roux, Lebo F. Gafane-Matemane, Sanette Brits, Johannes M. van Rooyen, Henner Hanssen, Ruan Kruger

**Affiliations:** ^1^Hypertension in Africa Research Team (HART), North-West University, Potchefstroom, South Africa; ^2^Medical Research Council (MRC) Research Unit for Hypertension and Cardiovascular Disease, North-West University, Potchefstroom, South Africa; ^3^Department of Sport, Exercise and Health, Medical Faculty, University of Basel, Basel, Switzerland

**Keywords:** arterial stiffness, central retinal artery equivalent, central retinal vein equivalent, arterio-venous ratio, ethnicity, pediatric

## Abstract

**Background:** Cross-talk between the macro-and microvasculature is considered an important contributor to target organ damage. Previous findings were predominantly in adult populations and investigation into this mechanism in children may provide insight into the development of early adverse vascular changes. Whether any ethnic differences in cross-talk is evident, also remains to be determined.

**Objective:** To determine whether retinal microvascular diameters are associated with large artery stiffness in young children and whether ethnic differences are evident.

**Materials and Methods:** In this cross-sectional study, 730 black (*n* = 437) and white (*n* = 293) school children aged 5-9 years were included. Pulse wave velocity (PWV) was measured and the central retinal arteriolar equivalent (CRAE) and central retinal venular equivalent (CRVE) diameters were calculated from fundus images. The arterio-venous ratio (AVR) was subsequently calculated.

**Results:** Pulse wave velocity was lower (*p* ≤ 0.001) in the black group when compared to the white group. The black group had a narrower CRAE, wider CRVE and lower AVR (all *p* < 0.001). Pulse wave velocity associated negatively with CRAE (*r* = –0.141, *p* = 0.003) and AVR (*r* = –0.185, *p* ≤ 0.001) in the black group only. A positive association between PWV and CRVE was seen in the black (*r* = 0.174, *p* ≤ 0.001) and white (*r* = 0.119, *p* = 0.043) group.

**Conclusion:** Large artery stiffness is associated with retinal arterial narrowing and venular widening in children, suggesting cross-talk between the macro-and microvasculature. Ethnic differences in these associations are also evident. Our findings warrant further investigation into environmental and sociocultural risk factors contributing to premature cardiovascular disease development.

## Introduction

Cardiovascular disease (CVD) remains a major health challenge globally. This challenge is not limited to adult populations as an increasing trend in hypertension and the subsequent future development of CVD in childhood populations has become evident (1–3). A number of cardiovascular risk factors track from childhood into adulthood which may initiate early onset of CVD and related mortality later in life (4, 5), highlighting the importance of primary and even primordial prevention of CVD (6).

The importance of large artery stiffness in cardiovascular (CV) risk prediction in adults is well established, with studies showing associations with end organ damage and clinical outcomes (7). Arterial stiffening is a natural consequence of aging and the associated natural biological deterioration of vascular structure and function, however in any given population some individuals are at higher risk of accelerated biological aging, placing them on a trajectory of early vascular aging (EVA) (8, 9). As central arterial stiffness is also considered one of the earliest detectable manifestations of vascular compromise (10), adverse changes in measures of arterial stiffness in children may provide insight into possible early vascular compromise and create opportunity for interventions.

In addition to the prognostic value of large artery stiffness parameters in cardiovascular risk prediction, the retinal microvasculature enables the investigation of the manifestation of systemic vascular diseases on a microvascular bed (11). Indeed, retinal microvascular alterations such as retinal arteriolar narrowing, venular widening and the resulting lower arteriolar to venular ratio (AVR) predict the risk of hypertension and stroke (12, 13) and is associated with cardiovascular mortality (14) in adults. It is proposed that cross-talk between large and small arteries occurs and that CV risk factors affect both vascular beds, even in children (15). Cross-talk between these vascular beds is also supported by the findings from a study including Swiss children (aged 6–8 years, *n* = 1171), where large artery stiffness, as measured by pulse wave velocity (PWV), was associated with retinal arterial narrowing as well as venular widening (15). Aside from these findings, data regarding cross-talk between large and small arteries in children is limited. An important factor of consideration is whether ethnic differences in the cross-talk between large and small arteries will be evident, as higher aortic stiffness has been extensively reported in black pediatric population groups compared with age-matched white groups independent of traditional risk factors (16–19). Data on ethnic differences in microvascular function in children is limited, however studies in adult populations have established that microvascular dysfunction is more evident in black groups when compared to their white counterparts (11, 20).

The investigation of preclinical measurements in children may provide novel insight into accelerated vascular deterioration, also coined EVA (21), a significant contributor to hypertension and CVD. We therefore aimed to determine whether retinal microvascular diameters are associated with large artery stiffness, as determined by PWV, in young children and whether ethnic differences in these associations are at play.

## Materials and Methods

The Exercise, Arterial Modulation and Nutrition in Youth South Africa (ExAMIN Youth SA) study was designed to investigate the interplay between body composition, dietary intake, physical fitness, and physical activity, psychosocial stress, cardiovascular function as well as urinary and salivary biomarkers. In this cross-sectional study we included data from 730 apparently healthy children (aged 5–9 years) that included black (*n* = 437) and white (*n* = 293) girls and boys, after the exclusion of participants with missing data for arterial stiffness and retinal vessel diameters (*n* = 332).

The study population and protocol for the ExAMIN Youth SA study has been described elsewhere (22). Briefly, children (aged Five to Nine years) of both sexes and all ethnicities attending public primary schools within two of the southern municipal areas of the Dr. Kenneth Kaunda district, namely JB Marks (Potchefstroom) and Matlosana (Klerksdorp) in the North West province, South Africa, were invited to participate voluntarily with parental permission. There were no specific exclusion criteria; however, children were excluded if no informed consent from the parent was obtained or if the child did not want to participate. On the day of participation, no children presented with any known illnesses.

The study was conducted in line with the ethical principles of the Declaration of Helsinki (23), was approved by the Health Research Ethics Committee of the North-West University and is registered at ClinicalTrials.gov (NCT04056377). All participants and their parents were fully informed about the objectives of the study and written informed consent/assent was obtained from each participant.

### Anthropometric Measures

All anthropometric procedures were performed according to specific guidelines set out by the International Society for the Advancement of Kinanthropometry (ISAK) (22, 24). Waist circumference (cm) was obtained in triplicate using standard protocol (Lufkin® Executive thin line 2 mm steel tape; Apex Tool Group B.V.; AK Emmen, Netherlands). The body mass index (BMI) [weight (kg)/square height (m^2^)] of each participant was calculated (SECA portable 213 stadiometer; SECA 813 electronic scale; Birmingham, UK). Body mass index z-scores and percentiles were calculated according to child growth reference data based on their age and sex (25).

### Cardiovascular Measures

#### Blood Pressure

Participants were required to remain in a relaxed chair-seated position for 3–5 min prior to blood pressure (BP) measurements. With the use of a validated automated oscillometric pediatric BP monitor (Omron HBP-1100-E; OMRON HealthCare Co., LTD. Kyoto, Japan) and the correctly sized BP cuff, brachial BP was measured with the participants' feet on the floor and their back and right arm supported (26, 27). Measurements were conducted five times with one-min intervals on the right arm (28, 29). The three measurements with the smallest variation were used to calculate a mean (30). Systolic blood pressure (SBP) and diastolic blood pressure (DBP) were captured from each measurement. Mean arterial pressure (MAP) was calculated using the following formula (DBP) + (0.4^*^pulse pressure) [35]. Prior to BP measurements, participants were also required to avert from using any stimulants (food and/or drugs).

#### Pulse Wave Analysis

Arterial pulse wave analysis was performed with the use of the validated oscillometric Mobilo-O-Graph monitor (I.E.M GmbH, Germany) and integrated ARCSolver software. Participants were in a seated position and using a correctly sized cuff on the mid-upper right arm. Measures of the central systolic- (cSBP) and diastolic blood pressure (cDBP), stroke volume, cardiac output, total vascular resistance, and arterial PWV were determined. Participant data was downloaded using the HMS Client-Server software package version 4.7.1 (I.E.M GmbH, Germany).

#### Retinal Vessel Analysis

Static retinal blood vessel images were captured using a Static Retinal Vessel Analyzer (SVA-T, Imedos Systems GmbH, Jena, Germany). The system consists of a fundus camera (Topcon TRC NW8) and analyzing software (Visualis 2.80, Imedos Systems GmbH, Jena Germany), allowing non-invasive and non-mydriatic assessment of retinal vessel diameters. Two valid images from the retina of both the left and right eye with an angle of 45° and with the optic disc in the center were taken per child. Retinal arterioles and venules, coursing through an area of 0.5–1 disc diameter from the optic disc margin, were semi-automatically identified at higher magnification using the Vesselmap 2, Visualis, Imedos Systems GmbH software. The examiner differentiated all retinal arterioles and venules in the outer ring-zone and measured them with the software tools. Vessel diameters were averaged to central retinal artery (CRAE) and vein equivalents (CRVE), using the ParrHubbard formula [41] and the arterio-venous ratio (AVR) was subsequently determined (CRAE/CRVE). For the CRAE and CRVE the mean of the right eye results was used.

### Statistical Analysis

For statistical analyses, IBM® SPSS® version 27 (IBM Corporation, Armonk, New York) and GraphPad Prism version 5.03 for Microsoft® Windows (GraphPad Software, San Diego, California, USA) were used to analyze and plot the data. Variables were tested for normality using the Kolmogorov-Smirnov test and QQ-plots. Data was expressed as mean ± standard deviation.

For comparisons between the groups, independent *t*-tests were used. Analysis of covariance was also used to determine differences in brachial blood pressure measures with adjustments for age, sex, and body height. Pulse wave velocity was additionally adjusted for MAP while CRAE was adjusted for CRVE and vice versa. Pearson and partial correlations (adjusted for age, sex and MAP) were used to determine the relationships of large artery stiffness with retinal vessel calibers.

## Results

The general characteristics of the study population, stratified by ethnicity are presented in Table 1. The ethnic groups were comparable in terms of age (*p* = 0.96), while the black group had lower body height, body weight and standardized BMI (all *p* ≤ 0.001) when compared to the white group. The black group presented with higher brachial DBP (*p* ≤ 0.001) while the central blood pressure measures were comparable (all *p* ≥ 0.36). In terms of vascular function, the black group showed a higher total vascular resistance (*p* ≤ 0.001), while PWV was lower (*p* ≤ 0.001) when compared to the white group. The black group had a narrower CRAE, wider CRVE and subsequently a lower AVR (all *p* ≤ 0.001).

**Table 1:** General characteristics of children stratified according to ethnicity.

	**Black children**	**White children**	***p***
	**(*n =* 437)**	**(*n =* 293)**	
Age (years)	7.46 ± 0.967	7.58 ± 0.809	0.96
Sex, boys (*n %*)	213 (42.0)	163 (52.6)	**0.003**
**Body composition**			
Waist circumference (cm)	53.16 ± 5.86	58.3 ± 7.89	**<0.001**
Body height (cm)	121 ± 7.46	127 ± 6.99	**<0.001**
Body weight (kg)	23.6 ± 5.50	27.1 ± 6.48	**<0.001**
BMI (kg/m^2^)	15.9 ± 2.40	16.7 ± 6.48	**<0.001**
BMI z-score	−0.182 (−2.13; 1.75)	2.41 (−1.43; 2.06)	**<0.001**
**Cardiovascular measures**			
Brachial systolic blood pressure (mmHg)^*^	101 ± 10	104 ± 10	0.44
Brachial diastolic blood pressure (mmHg)^*^	65 ± 8	64 ± 7	**<0.001**
Brachial mean arterial pressure (mmHg)^*^	80 ± 8	80 ± 7	**0.001**
Central systolic blood pressure (mmHg)	96 ± 10	96 ± 8	0.61
Central diastolic blood pressure (mmHg)	65 ± 8	65 ± 8	0.36
Pulse wave velocity (m/s)^†^	4.42 ± 0.328	4.53 ± 0.295	**<0.001**
Stroke volume (ml)	46.9 ± 7.99	51.2 ± 9.56	**<0.001**
Cardiac output (L/min)	4.10 ± 0.557	4.37 ± 0.646	**<0.001**
Total vascular resistance (mmHg/ml/s)	1.23 ± 0.133	1.19 ± 0.153	**<0.001**
**Retinal calibers**
Central retinal artery equivalent (MU)^‡^	198 ± 14.1	200 ± 15.0	**<0.001**
Central retinal vein equivalent (MU)^‡^	241 ± 15.2	230 ± 15.6	**<0.001**
Arterio-venous ratio	0.825 ± 0.063	0.870 ± 0.057	**<0.001**

*Values are arithmetic mean ± standard deviation or geometric mean (5th and 95th percentiles) for logarithmically transformed variables*.

*Bold values denote statistical significance (p < 0.05)*.

* *Brachial blood pressure measures were adjusted for age, sex, and body height*.

† *Pulse wave velocity was adjusted for age, sex, and mean arterial pressure*.

‡ *Central retinal artery equivalent was additionally adjusted for central retinal vein equivalent and vice versa*.

*n, number of participants; MU, measuring units*.

**Figure 1:**
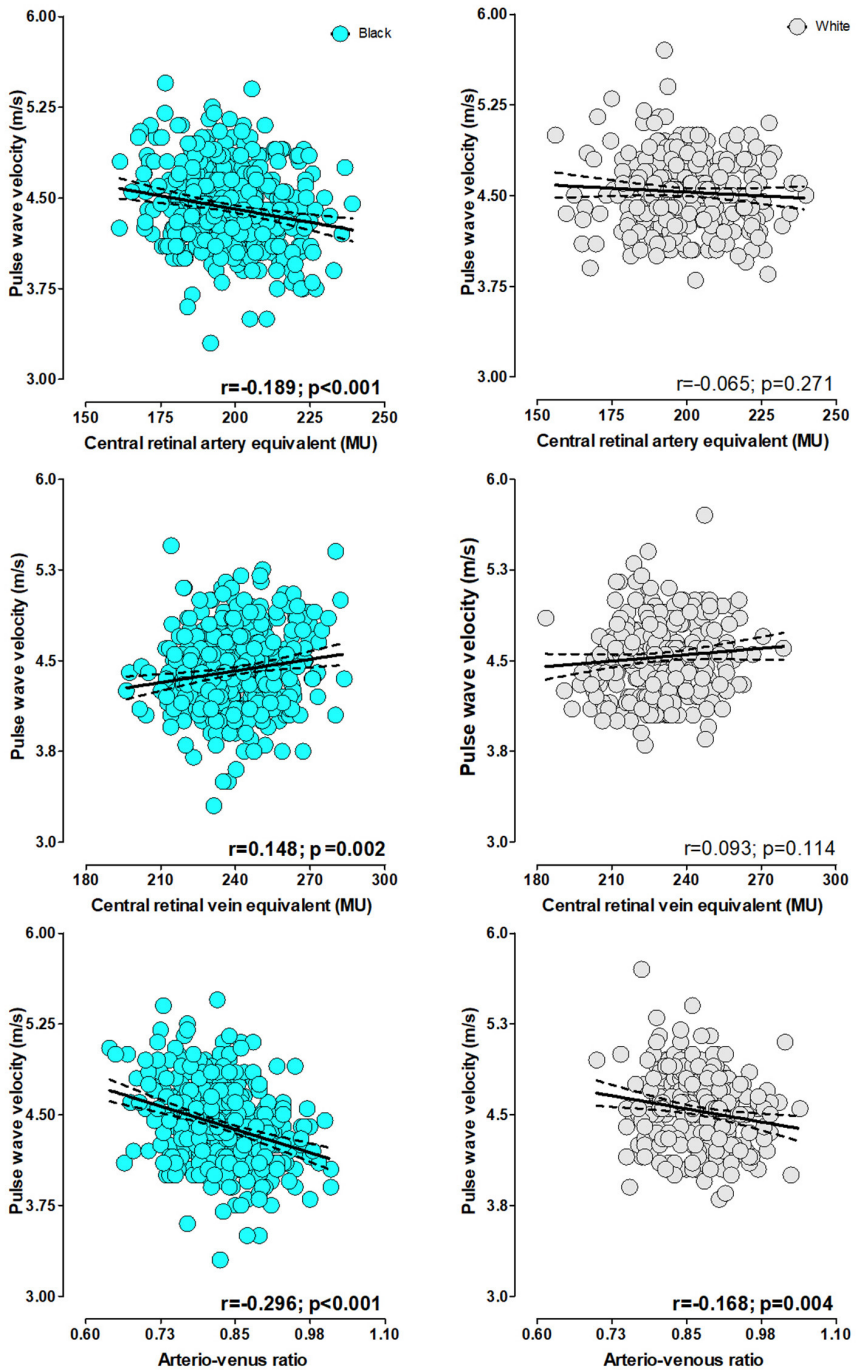
Single regression analyses pulse wave velocity with retinal calibers stratified by ethnicity.

In single regression analyses (Figure 1), PWV associated negatively with CRAE (*r* = –0.189, *p* ≤ 0.001) and positively with CRVE (*r* = 0.148, *p* = 0.002) in the black group only. A negative association between PWV and AVR was evident in the black (*r* = –0.296, *p* ≤ 0.001) and the white group (*r* = –0.168, *p* = 0.004).

The results from the single regression analyses were confirmed in partial regression analyses (Table 2) with adjustments for age and sex and MAP. PWV associated negatively with CRAE (*r* = –0.141, *p* = 0.003) and AVR (*r* = –0.185, *p* ≤ 0.001) in the black group only. In addition, a positive association between PWV and CRVE was also observed in the black group (*r* = 0.174, *p* ≤ 0.001). In the white group, PWV associated positively with CRVE (*r* = 0.119, *p* = 0.043).

**Table 2:** Partial correlations between large artery stiffness and retinal calibers in children stratified according to ethnicity.

	**Central retinal artery equivalent (MU)**		**Central retinal vein equivalent (MU)**		**Arterio-venous ratio**	
	**Black**	**White**	**Black**	**White**	**Black**	**White**
	**(*n =* 437)**	**(*n =* 293)**	**(*n =* 437)**	**(*n =* 293)**	**(*n =* 437)**	**(*n =* 293)**
Pulse wave velocity (m/s)	***r** **=*** –**0.141;** ***p** **=*** **0.003**	*r =* −0.055; *p =* 0.348	***r** **=*** **0.174;** ***p** **<*** **0.001**	***r** **=*** **0.119;** ***p** **=*** **0.043**	***r** **=*** –**0.185;** ***p** **<*** **0.001**	*r =* −0.090; *p =* 0.125

*Variables included in the models were age and sex. Pulse wave velocity was adjusted for mean arterial pressure. Central retinal artery equivalent was additionally adjusted for central retinal vein equivalent and vice versa*.

*Bold values denote statistical significance (p < 0.05)*.

## Discussion

We aimed to investigate the cross-talk between large and small arteries in 730 primary school children by determining whether retinal microvascular calibers are associated with PWV. We further aimed to establish whether any ethnic differences in these associations are evident. Measures of microvascular function were more adverse in the black children, with this group showing a narrower CRAE, wider CRVE, and subsequently a lower AVR. In terms of macrovascular function, we found against expectations, that PWV was lower in the black children when compared to their white counterparts. Ethnic differences in the associations between retinal microvascular calibers and large artery stiffness were also evident and our most prominent finding was that large artery stiffness was associated with retinal arterial narrowing and venular widening in the black group, independent of age, sex, and MAP. In the white children, large artery stiffness was associated with venular widening only.

The concept of large and small artery cross-talk is nested in findings that showed a strong relationship between arterial stiffness and microvascular damage in various organs such as the heart, brain, retina, and kidneys (31–33). Cross-talk is described as a vicious circle of events with adverse changes such as increased wall-lumen ratio and rarefaction of small arteries (22, 34) driving an increase in blood pressure; which in turn, increases large artery stiffness through the process of vascular remodeling. Ultimately, increased large artery stiffness is a major determinant of increased pulsatile pressure, which damages small arteries (23) and favors the development of target organ damage (24). Data to support this concept has largely been published from studies including adult populations, especially within the setting of hypertension (25). The negative association between CRAE and PWV in the black group, as well as the positive association between CRVE and PWV in both ethnic groups in our study is suggestive of cross-talk between different vascular beds. Our results are in line with the findings from Salvetti et al. where a positive association between the wall-lumen ratio of retinal arterioles and PWV was reported in an adult cohort which included treated and non-treated hypertensive individuals (26). More recently, it was shown that the progression of microvascular dysfunction was associated with higher PWV after 4 years in a pediatric population (27), while a significant but weak inverse association between CRAE and PWV was also reported in a Swiss cohort of children aged 6–8 years (15).

In terms of the possible contribution of ethnicity to differences observed in cross-talk between the macro- and microvasculature, the black children also had a narrower CRAE and wider CRVE. Although data regarding ethnic differences in microvascular function is limited, our results are in line with those of a study that included young black and white adults (20-30 years) that also showed smaller CRAE values in the black group compared to their white counterparts, taking into account that the 24 h BP and anthropometric profiles were similar. Furthermore, data from the Atherosclerosis Risk in Communities Study (28) showed that black participants tended to have narrower retinal arteries and wider retinal venules, although this study included older individuals. Against expectations, in our study PWV was lower in the black children when compared to the white children. Our results contradict the findings of a number of previous adult studies that showed black population groups to have increased large artery stiffness, as measured by various markers such as PWV (20), pulse pressure amplification (29), and augmentation index (30). Despite the lower PWV observed in the black children, the associations between the macro-and microvascular parameters were more pronounced, with all retinal vessel calibers showing adverse associations with PWV in this group. These findings may infer that even at lower levels of arterial stiffness, cross-talk between these vascular beds is more significant in black children. A further important finding was the higher total vascular resistance observed in the black children, as it is known that vascular resistance in the large arteries is closely associated with the progression of arterial stiffness. Recent findings have also suggested that an increase in BP in childhood seems to be driven by peripheral resistance resulting in increased arterial stiffness and at a later stage; the manifestation of high BP might be mediated by arterial stiffness. Although numerous ethnic differences in arterial stiffness and microvascular function have been previously reported, whether ethnicity in itself can be regarded as a risk factor for EVA remains debatable. It is likely that multiple differences in environmental and sociocultural risk factors which may adversely influence biological aging could explain these differences and warrants further investigation.

This study must be interpreted within the context of its strengths and limitations. This study is limited by its cross-sectional design; hence cause and effect cannot be inferred. We only included children from the North West Province of South Africa and our sample may therefore not be representative of the population of the entire country. Although PWV was measured by brachial oscillometry, which is a method that has not been validated in pediatric populations, it has previously been associated with CV risk in children. This study was the first to report associations between PWV and retinal microvascular calibers in a large sample of children including different ethnic groups. For retinal vessel imaging, duplicate images were taken from the eye, allowing for a high accuracy in retinal vessel diameter detection. To expand on the present findings, future studies are warranted to determine differences in environmental and sociocultural risk factors in children from a multi-ethnic point of view that may ultimately impact EVA differently among ethnic groups. Moreover, our findings necessitate the screening of black pediatric subjects for early signs of vascular deterioration as well as determining the impact of lifestyle behaviors (physical fitness/activity, dietary intake, and psychosocial factors) involved in early vascular aging. Such data could aid in the aid in the development of primary prevention programs.

In conclusion, large artery stiffness is associated with retinal arterial narrowing and venular widening in children, with these findings being more pronounced in black children. Our findings suggest that cross-talk between the large and small arteries are already evident at an early stage in the life course and may provide insight into the development of early vascular aging. These results warrant further investigation into environmental and sociocultural risk factors contributing to premature cardiovascular disease development.

## Data Availability Statement

The raw data supporting the conclusions of this article will be made available by the authors, without undue reservation.

## Ethics Statement

The studies involving human participants were reviewed and approved by Health Research Ethics Committee of the North-West University. Written informed consent to participate in this study was provided by the participants' legal guardian/next of kin.

## Author Contributions

RK and HH conceptualized and designed the study. YB and AC was responsible for data analysis and YB wrote the original draft. AC, WS, SB-L, LG-M, SB, JR, HH, and RK contributed to the interpretation of data and critical review of manuscript. All authors gave final approval of the version to be submitted.

## Funding

This research funded in this manuscript is part of an ongoing research project financially supported by the South African Medical Research Council (SA MRC) Extra Mural Unit and the National Research Foundation (NRF) of South Africa for Competitive Support for Y-Rated Researchers (Unique Identification No: 112141), the NRF Equipment Related Training and Travel Grant (Unique Identification Number: 109905) and the South African Research Chairs Initiative (SARChI) of the Department of Science and Technology and National Research Foundation (NRF) of South Africa (Unique Identification No: 86895). Research reported in this article was supported by the South African Medical Research Council under a Self-Initiated Research Grant. In addition, we would like to thank International Atomic Energy Agency (IAEA) for financial support.

## Author Disclaimer

The views and opinions expressed are those of the author(s) and do not necessarily represent the official views of the SA MRC. Any opinion, findings and conclusions or recommendations expressed in this material are those of the authors, and therefore, the NRF does not accept any liability in this regard. None of the funding bodies contributed to the conceptualization, data collection or writing of this article.

## Conflict of Interest

The authors declare that the research was conducted in the absence of any commercial or financial relationships that could be construed as a potential conflict of interest.

## Publisher's Note

All claims expressed in this article are solely those of the authors and do not necessarily represent those of their affiliated organizations, or those of the publisher, the editors and the reviewers. Any product that may be evaluated in this article, or claim that may be made by its manufacturer, is not guaranteed or endorsed by the publisher.
